# Caregiver-reported barriers to care for children and adults with Williams Syndrome

**DOI:** 10.1007/s12687-024-00707-w

**Published:** 2024-05-22

**Authors:** Elizabeth W. Barnhardt, Marilee Martens, Rebecca Andridge, Jennifer Walton

**Affiliations:** 1 Section of Developmental and Behavioral Pediatrics, Nationwide Children’s Hospital, 380 Butterfly Gardens Drive, Columbus, OH USA; 2 Department of Psychology, Nationwide Children’s Hospital, 380 Butterfly Gardens Drive, Columbus, OH USA; 3 Division of Biostatistics, The Ohio State University College of Public Health, 242 Cunz Hall, Columbus, OH USA; 4 Section of Developmental and Behavioral Pediatrics, Department of Pediatrics, and Mailman Center for Child Development, University of Miami Miller School of Medicine, and Holtz Children’s Hospital, The Ohio State University College of Public Health, Jackson Health System, Miami, FL USA

**Keywords:** Williams syndrome, Barriers to care, Developmental disabilities

## Abstract

Individuals with Williams syndrome (WS) may experience a variety of medical, behavioral, and educational concerns. The primary objective of this study was to assess barriers to health care for patients with WS, primarily using the Barriers to Care Questionnaire (BCQ), and to assess whether various demographic factors are correlated with these barriers. A REDCap survey was distributed using the Williams Syndrome Association Research Registry. 319 caregivers of individuals with WS in the United States completed the BCQ. On the BCQ, lower scores indicate more barriers to care. Younger age was associated with lower scores for both the pragmatics and the skills subscales while lower income levels and increased distances to providers knowledgeable about WS were consistently associated with lower total BCQ scores.

**Supplementary Information:**

The online version contains supplementary material available at 10.1007/s12687-024-00707-w.

## Introduction

Williams syndrome (WS) is one of the most common microdeletion syndromes occurring in approximately 1 in 7500 persons and is caused by the loss of 26–28 contiguous genes mapping to chromosome 7q11.23 (Strømme et al. 2002). WS is a multisystem disorder that typically is identified in early childhood, presenting with characteristic facial features, hyper-social behavior, cardiovascular disease, short stature, connective tissue anomalies, hearing loss, endocrine abnormalities, developmental delays, and an increased prevalence of intellectual disability (Morris & Williams Syndrome 1999). In addition to the need for ongoing follow-up with multiple subspecialties, individuals with WS typically require early, intensive developmental support and ongoing educational accommodations. Individuals with WS are also at an increased risk for behavioral and mental health challenges including anxiety disorders and attention deficit-hyperactivity disorder (Woodruff-Borden et al. 2010; Leyfer et al. 2006). The unique medical, developmental, and behavioral supports needed for this population make it an important group to examine in regard to health care access and quality.

A significant amount of recent literature has focused on the identification of disparities with regard to health care access and quality for individuals with intellectual and developmental disabilities. Previously reported barriers include poor access to quality health care services, increased prevalence of behavioral and mental health conditions, lower rates of health prevention and promotion, and higher levels of adversity, especially in children with disabilities (Krahn et al. 2006; Berg et al. 2019). For example, in a secondary analysis of data on Adverse Family Experiences (AFEs) collected as part of the 2011–2012 National Survey of Children’s Health, Berg et al. (2019) found that children with developmental disabilities experienced a higher number of AFEs, in comparison to their typically developing peers. In this nationally representative survey, the Adverse Family Experiences questionnaire was created as an adaptation of the original Adverse Childhood Experiences questionnaire and asked about aspects of adversity children may be exposed to such as economic hardship, death of a parent, neighborhood violence, and discrimination based on race/ethnicity (Berg et al. 2019) There also exists an interdependent relationship between disability status and social determinants of health, exacerbating disparities in this population (Frier et al. 2018). Families have additionally reported difficulties in locating primary care providers (PCPs) with expertise serving individuals with intellectual and developmental disabilities and navigating complex systems of care that also include involvement with multiple medical subspecialists (Krahn et al. 2006). It should be noted that for some individuals with intellectual and developmental disabilities, several racial, ethnic, and cultural disparities have also been identified that exist regardless of income, health insurance, and access to care, resulting in an amplification of health disparities for individuals with intellectual and developmental disabilities from marginalized racial and ethnic groups (Yee et al. 2018; Scott and Havercamp 2014). Specifically, children from low-income and racial/ethnic minority backgrounds are identified later and with more severe symptoms, and they have been shown to receive less evidence-based treatment in comparison to affluent white children (Magaña et al. 2012).

For children with rare genetic syndromes, their parents have been noted to experience a significant amount of psychological distress related to diagnostic and prognostic uncertainty, and receipt of a specific diagnosis appears to be highly beneficial to the well-being of the family unit (Lenhard et al. 2005; Lewis et al. 2010). Previously, research has demonstrated an average age of diagnosis of WS of 3.66 years (Huang et al. 2002). There is also some evidence that features may differ in non-white individuals and contribute to a delay in diagnosis (Gold et al. 2021). With improved access to genetic testing over the past two decades, it is possible that this age of diagnosis will continue to decline. Individuals with WS are rarely identified at birth due to less pronounced facial features and may only be diagnosed in the newborn period if characteristic cardiac lesions, such as supravalvular aortic stenosis, or classic findings, such as hypercalcemia, are present, prompting genetic evaluation (Waxler et al. 2013; Shah et al. 2008). Otherwise, children may be diagnosed later in life when they present for subspecialty evaluation in the context of developmental delays or growth concerns (Shah et al. 2008).

Previous literature has indicated that the diagnostic process for parents of children with WS can be incredibly stressful with 59.91% of parents citing negative recollections of their experience (Waxler et al. 2013). Although a young child and/or adult may receive developmental and medical supports in the absence of a diagnosis, identification of a specific diagnosis has many potential benefits. In addition to the psychological benefit of diagnostic certainty, other benefits for individuals with WS might include access to diagnosis-specific supports such as educational and family support services through the Williams Syndrome Association (WSA), access to innovative treatments such as clinical trials or new surgical techniques, the opportunity to meet with experts at one of the ten Williams syndrome specialty clinics in the US, and the ability to participate in a community of support. In a recent study conducted by the Polish Williams Syndrome Association, families expressed dissatisfaction with the way the healthcare system for WS children works and complained about the doctors’ lack of knowledge about WS as well as a lack of access to specialist care (Domaradzki and Walkowiak 2024) There has been no similar study conducted with on the experiences of children and adults with WS in the US.

The purpose of this project is to examine barriers to health care for children and adults with WS, primarily using a validated tool, the Barriers to Care Questionnaire (BCQ). In this project, we had three guiding research questions:

1. What are the most commonly identified barriers to care for children and adults with Williams syndrome?
2. Which demographic factors are most highly associated with each barrier to care?
3. For individuals with Williams syndrome, what are their parents’ perceptions of the medical care which they receive from their primary care provider?

The BCQ has been validated in a vulnerable group of children with asthma (Seid et al. 2009) and a generalized population of children with special healthcare needs (Seid et al. 2004) and has also been utilized in other groups of children with special healthcare needs (Razdan et al. 2019; Bennett et al. 2018; Sadreameli et al. 2018; Jacob et al. 2016; Cassell et al. 2014). The BCQ uniquely conceptualizes barriers to care as affecting processes of care, labelling items such as race and socioeconomic status as proxies for other processes, rather than explanatory variables themselves (Seid et al. 2004). Previous literature has attempted to describe the barriers faced with regard to access and quality of care for populations of individuals with similar developmental disabilities (Gilbertson et al. 2019; Ploeg Booth 2011; Bilaver and Havlicek 2019), however, no study has evaluated the specific barriers to care for the WS population.

In our study, we also sought to assess whether demographic factors are correlated with these barriers and quantify therapy utilization within the WS population. Additionally, we sought to characterize the primary care experiences of individuals with WS. By understanding the barriers to care in this unique population, we hope to shed light on how best to create programs that support children and adults with rare genetic syndromes and develop systems of care that emphasize quality and improved access.

## Materials and methods

### Participants

An online survey was designed using REDCap (Research Electronic Data Capture) and distributed to 1,289 caregivers of individuals with WS and adults with WS via the Williams Syndrome Association Research Registry. Participants were incentivized by being entered to win one of two $50 visa gift cards upon completion of the survey. Participant email addresses were obtained and used to distribute REDCap is a browser-based software program used for designing clinical and translational research databases (Harris et al. 2009). The survey was anonymous but did request an email address for future gift card distribution. This data was stored separately from survey results. Study inclusion criteria required participants to have a clinical or genetic confirmation of WS as verified by parent or caregiver. After an initial email was sent containing the link to the study, a postcard was designed and mailed to all members of the registry who provided home addresses along with email contact information. Additionally, 200 postcards were distributed at a Williams Syndrome Association National Convention in 2018. The survey was available for completion for a six month period to include 3 months prior to and 3 months following the convention.

### Survey design

The survey included demographic and socioeconomic information collected via parent or caregiver report, and respondents were asked about healthcare service utilization as well as primary care experiences. Parents were also asked to estimate their distance to a provider they deem extremely knowledgeable about Williams syndrome (also known as a WS provider). Parents and caregivers were also asked to provide free text responses discussing their biggest struggles in obtaining medical care for their child. The complete survey to include instructions provided to families is included as an appendix.

Our primary study measure was the 39-item Barriers to Care Questionnaire (BCQ) (Seid et al. 2004), which is a validated tool to assess health care barriers, developed through literature review, focus groups, and interviews with Spanish- and English-speaking parents of children with chronic health conditions. The BCQ uses parent report to measure barriers to care using five different scales described below. It yields a 0 to 100 score, with higher scores denoting fewer barriers and lower scores denoting increased barriers to care for the total scale and subscales. The reading level of the BCQ is at a 5.7 grade level as assessed by the Flesch-Kincaid readability scale (Seid et al. 2004). The BCQ subscales include the following:

1. Pragmatics: logistical and cost issues that might prevent or delay appropriate utilization (9 items).
2. Skills: acquired or learned strategies to navigate through, manipulate, or function competently within the health care system (8 items).
3. Expectations: parent expectations of receiving poor-quality care (7 items).
4. Marginalization: the internalization and personalization of negative experiences within the health care system (11 items).
5. Knowledge and beliefs: lay or popular ideas about the nature and treatment of illness, which may differ from those of mainstream allopathic medicine. (4 items)

The BCQ has the following instructions: “Parents often face barriers when trying to get health care for their children. We are interested in the kinds of things that interfere with getting health care for your child(ren). Please rate how much of a problem each of the following is for you.” Response scores and choices were 100 for “No Problem,” 75 for “Small Problem,” 50 for “Problem,” 25 for “Big Problem,” and 0 for “Very Big Problem”. We were unable to find guidelines as to how to handle missing items in the BCQ subscales. Thus we adopted the following strategy: if at most one item was missing in a subscale, compute the subscale score as the average of the answered items (equivalent to replacing the missing item with the mean of the other questions in the subscale). If more than one item was missing in a subscale, do not compute a subscale score. The threshold of only one missing item was chosen since one subscale (Knowledge and Beliefs) only had four items.

### Statistical methods

Descriptive statistics were calculated to describe the study sample. Scores on the BCQ were left-skewed and thus were summarized using both means and medians. Cronbach’s alpha was calculated as a measure of internal consistency for each of the BCQ subscales and the total score. ANOVA was used to test for differences in mean BCQ scores by demographic factors and Pearson’s correlation was used to quantify the relationship between BCQ scores and age. Due to the skewness of BCQ scores, analyses were repeated using Kruskal-Wallis nonparametric ANOVA and Spearman’s correlation; conclusions were similar and are not shown. All predictors significant at the *p* < .05 level were then simultaneously entered into adjusted linear regression models for BCQ (one for each subscale and the overall score). Due to sporadic missing data for demographic data, not all analyses used the entire analysis sample. All analyses were conducted using SAS version 9.4 (Cary, NC) and R (R Core Team, 2020).

This project was reviewed and approved by the Institutional Review Board at Nationwide Children’s Hospital and the Williams Syndrome Association Research Registry.

## Results

### Analysis sample

A total of 368 individuals completed or partially completed the survey. The survey did not require participants to specify how they learned about the survey (either from the postcards sent to members of the WSA Research Registry or the WSA National Convention), thus a response rate could not be calculated. However, it is estimated that less than 50 respondents were recruited specifically from the Convention based on the timing of completion of the survey responses. Of the 368 completed or partially completed surveys, 49 surveys (13%) were excluded from all analyses due to missing data: 37 surveys with all BCQ questions missing, six with at least one of the BCQ subscales missing, and six with more than one item missing within at least one subscale (not able to be scored). This resulted in 319 participants in the analysis sample.

### Demographic summary

The individuals with WS were relatively demographically diverse (Table 1). The mean age was 18.2 (SD = 12.9), with a range from 0 to 65.8 years. The majority of individuals with WS were diagnosed at a young age (median age at diagnosis = 11 months) but there was considerable variability (range: 0 months to 32 years), and the majority were diagnosed via FISH (68.3%). It is likely we are seeing more individuals diagnosed via FISH due to the mean age of our sample (18.2 years) rather than more current methodologies often used for diagnosis such as a chromosomal microarray. It may be that our study demonstrated a younger median age of diagnosis than previously published studies due to the highly-engaged nature of our sample, which includes caregivers of individuals with Williams syndrome who are either highly involved with the Williams Syndrome Association or its research registry. The age of diagnosis in our sample may also reflect the more widespread availability of genetic testing and an increased awareness of the signs and symptoms which can be associated with Williams syndrome which warrant genetic evaluation. The sample contained roughly equal numbers of female and male individuals with WS and were predominantly white (82.6%) and English-speaking (97.5%). Participants represented diverse regions of the United States (US), with 24.4% of participants from the Midwest Region, 29.5% from the Northeast, 19.2% from the Southeast, 10.1% from the Southwest, and 16.9% from the West. More than half the participants lived in a suburban setting (62.9%) but there was some representation from both urban (15.2%) and rural (21.9%) areas. The caregivers who responded tended to be well-educated, with 73% reporting that at least one caregiver in the household had a bachelor’s degree or higher, and 47.3% reported an annual household income of over $100,000.

**Table 1:** Demographic summary of 319 participants

Characteristic	*N* (%) or Mean (SD)
Current age, years (*n* = 304)	
Mean (SD)	18.2 (12.9)
Median (25th, 75th percentile)	15.4 (8.3, 26.7)
Range	0.0 to 65.8
Age at diagnosis, months (*n* = 273)	
Mean (SD)	24.5 (43.2)
Median (25th, 75th percentile)	11 (3, 23)
Range	0 m to 32 y
Method of Diagnosis*	
FISH	218 (68.3%)
Microarray	64 (20.1%)
Clinical evaluation	98 (30.7%)
Sex	
(0) Female	167 (53.0%)
(1) Male	148 (47.0%)
Race	
(1) White	262 (82.6%)
(2) Black or African American	10 (3.2%)
(3) Asian/Pacific Islander	7 (2.2%)
(4) Hispanic or Latino	25 (7.9%)
(5) Other	13 (4.1%)
Language	
(1) English	309 (97.5%)
(2) Spanish	5 (1.6%)
(3) Arabic	1 (0.3%)
(5) Other	2 (0.6%)
Region	
(1) Midwest	75 (24.4%)
(2) Northeast	91 (29.5%)
(3) Southeast	59 (19.2%)
(4) Southwest	31 (10.1%)
(5) West	52 (16.9%)
Location	
(1) Urban	48 (15.2%)
(2) Suburban	198 (62.9%)
(3) Rural	69 (21.9%)
Insurance	
(1) Medicaid	100 (32.2%)
(2) Private	191 (61.4%)
(3) Other/Self-Pay	20 (6.4%)
Household Income	
(1) Less than $20,000	9 (3.2%)
(2) $20,000 to $34,999	16 (5.7%)
(3) $35,000 to $49,999	16 (5.7%)
(4) $50,000 to $74,999	57 (20.3%)
(5) $75,000 to $99,999	50 (17.8%)
(6) Over $100,000	133 (47.3%)
Highest Education in Household	
(1) < High School	3 (0.9%)
(2) High School Diploma/GED	18 (5.7%)
(3) Some college (no degree)	30 (9.4%)
(4) Trade/technical/vocational training	16 (5.0%)
(5) Associate’s degree	19 (6.0%)
(6) Bachelor’s degree	110 (34.6%)
(7) Master’s degree	88 (27.7%)
(8) Professional degree	12 (3.8%)
(9) Doctorate degree	22 (6.9%)

*Respondents could endorse more than one option

Missing data: sex: *n* = 4; race: *n* = 2; region: *n* = 11; location: *n* = 4; language: *n* = 2; insurance: *n* = 8; household income: *n* = 38; highest education in household: *n* = 1

Table 2 summarizes parents’ perceptions of the medical care they receive from their primary care provider as well as other provider-related characteristics. Nearly three-quarters of the sample lived within 30 miles of a pediatric ER, but only 30% lived within 30 miles of a WS provider (a provider deemed extremely knowledgeable about Williams syndrome). More than half the sample (37.5%) lived more than 90 miles from a WS provider. Almost all individuals had a primary care physician (PCP) in the last year and the vast majority had seen their PCP in the past year. However, only 14.8% reported that their PCP was extremely knowledgeable about WS; 40.2% rated their PCP as having limited or no knowledge of WS.

**Table 2:** Summary of provider-related and WS-knowledge-related characteristics

Characteristic	*N*%
Distance to closest WS Provider	
(1) < 30 miles	93 (30.1%)
(2) 30–60 miles	53 (17.2%)
(3) 60–90 miles	47 (15.2%)
(4) > 90 miles	116 (37.5%)
Distance to closest Pediatric ER	
(1) < 30 miles	226 (72.4%)
(2) 30–60 miles	46 (14.7%)
(3) 60–90 miles	17 (5.4%)
(4) > 90 miles	23 (7.4%)
Has PCP	
(0) No	5 (1.6%)
(1) Yes	313 (98.4%)
Has PCP Visit in Last Year	
(0) No	13 (4.1%)
(1) Yes	303 (95.9%)
PCP’s Knowledge About WS	
(1) Extremely knowledgeable	47 (14.8%)
(2) Somewhat knowledgeable	143 (45.0%)
(3) Limited knowledge	112 (35.2%)
(4) No knowledge	16 (5.0%)
Caregiver’s Confidence Explaining to a Provider Unfamiliar with WS	
Medical Aspects of Child’s Care	
(1) Very confident	205 (64.5%)
(2) Somewhat confident	104 (32.7%)
(3) Not confident at all	9 (2.8%)
Their Child’s Behavioral/Emotional Care	
(1) Very confident	207 (64.9%)
(2) Somewhat confident	98 (30.7%)
(3) Not confident at all	14 (4.4%)
Their Child’s Learning/School Needs	
(1) Very confident	178 (58.0%)
(2) Somewhat confident	120 (39.1%)
(3) Not confident at all	9 (2.9%)

WS = Williams Syndrome; PCP = Primary Care Physician

Missing data: distance to WS provider: *n* = 10; distance to pediatric ER: *n* = 7; has PCP: *n* = 1; PCP knowledge about WS: *n* = 1; confidence explaining overall care: *n* = 1; confidence explaining school care: *n* = 12

We also summarized the therapies being received by young children with WS. Interestingly, the results demonstrate only 56% of children age 0–3 years in our sample receiving speech therapy through early intervention services. The data also demonstrates an unexpectedly low participation rate in school therapies, with only 14% of children ages 3–18 years receiving speech therapy in school, 10% receiving physical therapy in school, and 17% receiving in-school occupational therapy. 3% of children ages 3–18 were noted to be receiving music therapy in school and 3% of children from infant through 18 years were receiving private music therapy. Additional data on services received is provided in Appendix B.

### Barriers to care questionnaire

The mean overall BCQ score was 86.3 (SD 12.7), with a range from 27.5 to 100. Means for the subscales are shown in Table 3. As stated previously, the BCQ yields a 0 to 100 score, with higher scores denoting fewer barriers and lower scores denoting increased barriers to care for the total scale and subscales. The highest subscale scores (fewest barriers to care) were for the knowledge and beliefs subscale (mean = 92.5, SD = 12.2); more than half the sample (58.3%) had a score of 100, meaning they endorsed “no problem” for all items in this subscale. The lowest average subscale score was for pragmatics (mean = 80.4, SD = 17.4), where only 14.1% of the sample scored at the maximum value. The remaining subscales had averages around 84 to 88, with roughly 25–30% of the sample scoring at the maximum. Internal consistency was very high for the total score (α = 0.95) and for the subscales (α = 0.78 to 0.92).

**Table 3:** Summary of barriers to care scale

Scale	Mean (SD)	Median (IQR)	Range	*N* (%) at Max	Cronbach’s α*
Overall	86.3 (12.7)	89.4 (79.7–96.0)	27.5–100	18 (5.6%)	0.95
Skills	86.3 (14.6)	90.6 (78.1–96.9)	25–100	79 (24.8%)	0.83
Marginalization	88.3 (15.4)	93.2 (84.1–100)	11.4–100	93 (29.2%)	0.92
Expectations	84.1 (17.5)	89.3 (75.0-100)	10.7–100	81 (25.4%)	0.85
Knowledge and Beliefs	92.5 (12.2)	100 (87.5–100)	18.8–100	186 (58.3%)	0.78
Pragmatics	80.4 (17.4)	83.3 (69.4–94.4)	8.3–100	45 (14.1%)	0.84

SD = standard deviation; IQR = Interquartile range

**Fig. 1:**
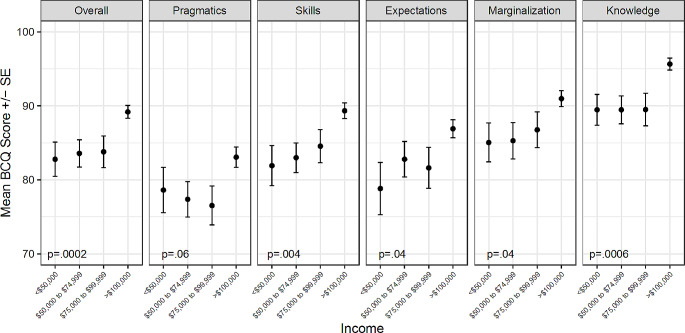
Mean BCQ overall and subscale scores by levels of income

**Fig. 2:**
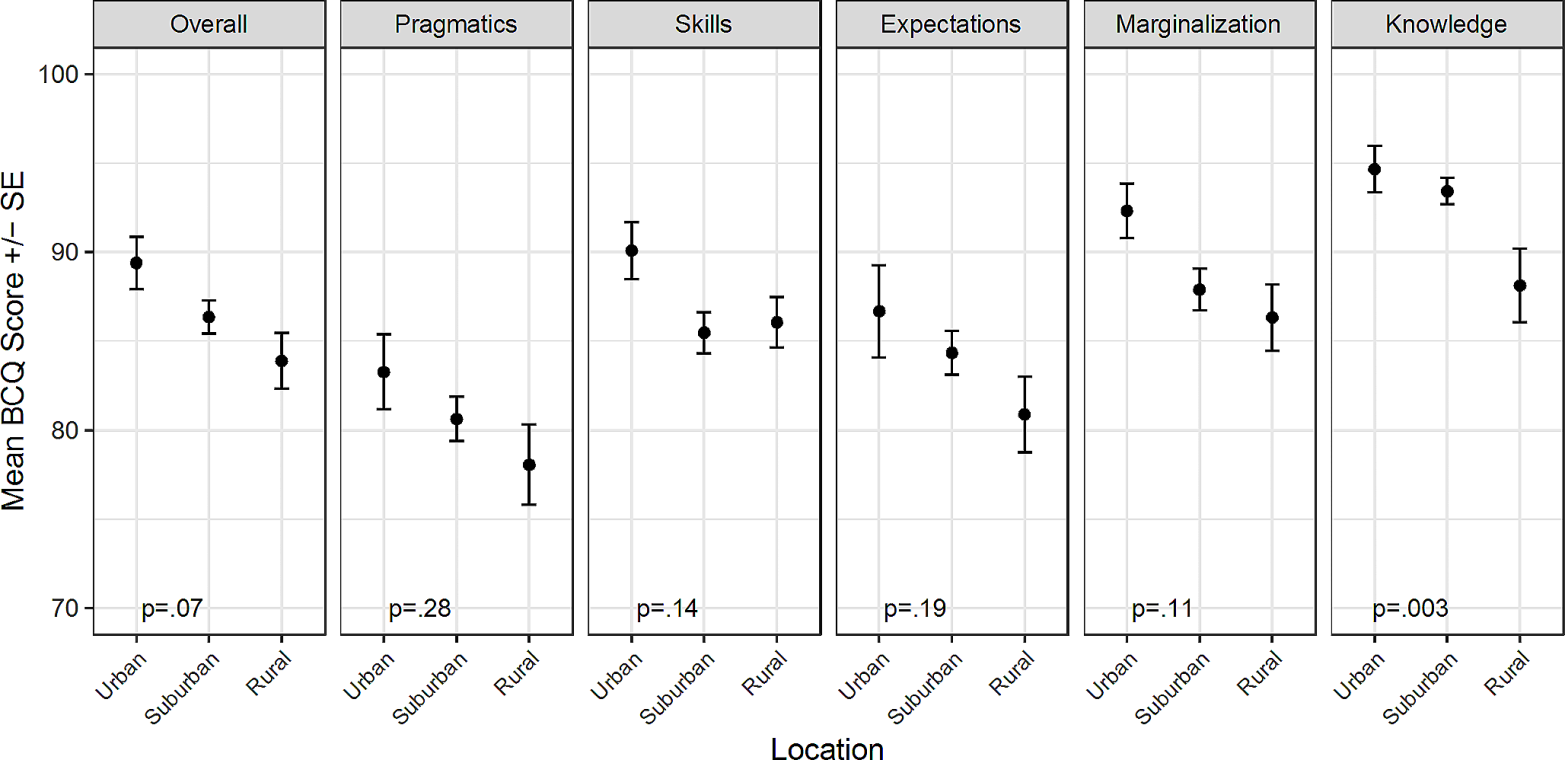
Mean BCQ overall and subscale scores by location

BCQ scores and subscale scores were statistically significantly associated with several demographic variables. There was a weak but statistically significant correlation between age and both the pragmatics subscale (*r* = .21, *p* = .0003) and the skills subscale (*r* = .13, *p* = .03), with older age associated with higher scores (fewer barriers). None of the other subscales or the overall BCQ score were associated with age. The overall BCQ score and all subscales except pragmatics were associated with income (*p* < .05 for all; Fig. 1). Individuals with the highest level of household income (>$100,000) had the highest mean BCQ scores (fewest barriers) while individuals with lower levels of income had lower and more similar mean scores. Additionally, there was a significant difference in mean BCQ knowledge scores by location, with individuals in rural areas having lower scores, indicating more barriers (*p* = .003; Fig. 2).

There were similar trends, though non-significant, for all other subscales and the overall score, with individuals in rural areas having the lowest scores and those in urban areas having the highest scores. There were no differences in BCQ overall or subscale scores based on the sex or race of the individual with WS, caregiver education level, insurance type, or region of the country (*p* > .05 for all). The demographic characteristics for overall and subscale BCQ scores are included in Appendix B.

**Fig. 3:**
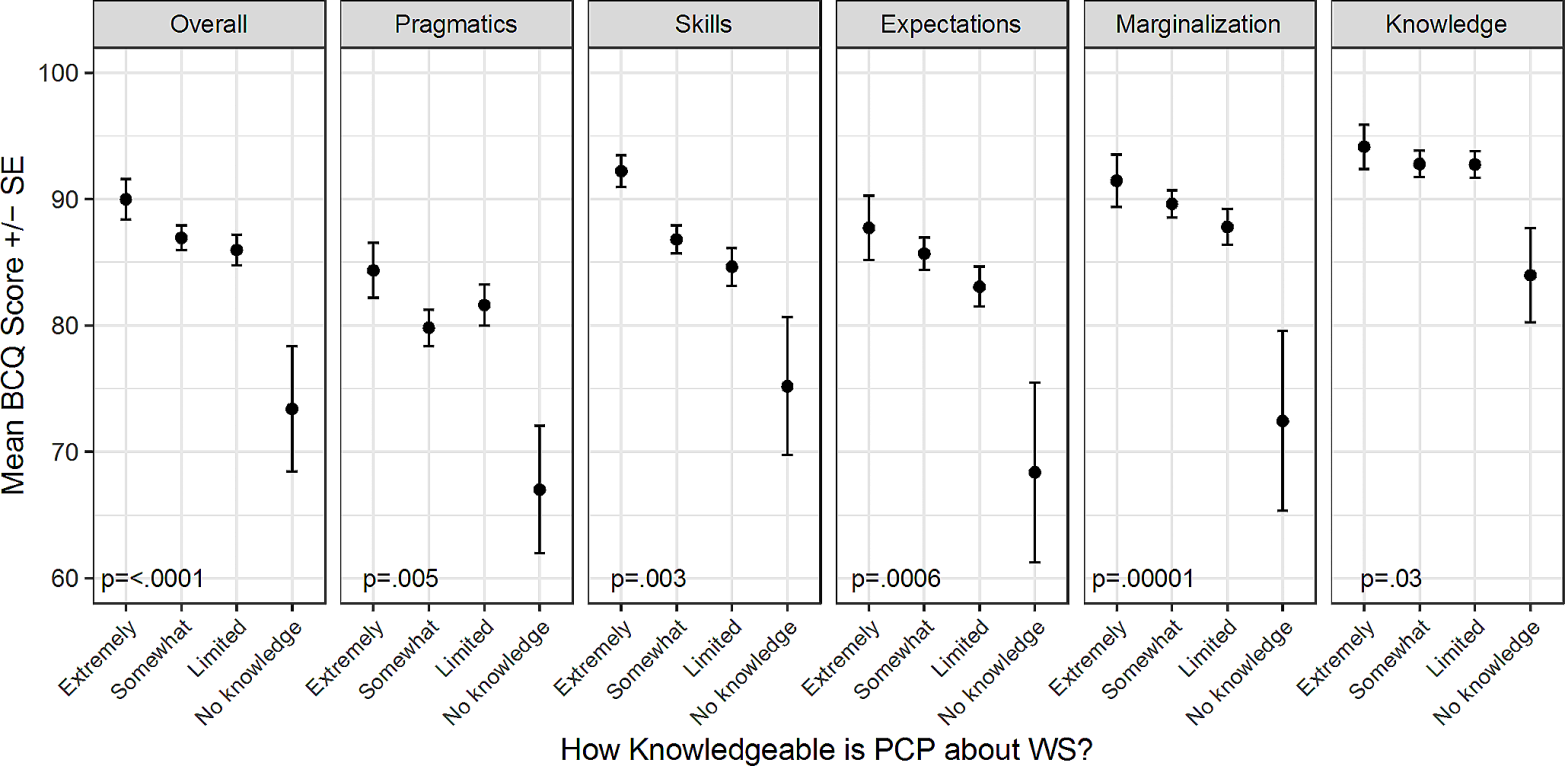
Mean BCQ overall and subscale scores by perceived PCP knowledge of WS

Lack of access to knowledgeable WS providers was significantly associated with more barriers to care, as indicated by lower BCQ overall and subscale scores. As shown in Fig. 3, there were significant associations between perceived PCP knowledge of WS and barriers to care for the overall BCQ score and all five subscales (*p* < .05 for all). Though the number of respondents reporting that their PCP had no knowledge of WS was small, their BCQ scores were significantly lower than the scores of participants who perceived their PCP to know more about WS. For most subscales there was a trend of increasing scores (decreasing barriers) as the perceived PCP knowledge increased.

**Fig. 4:**
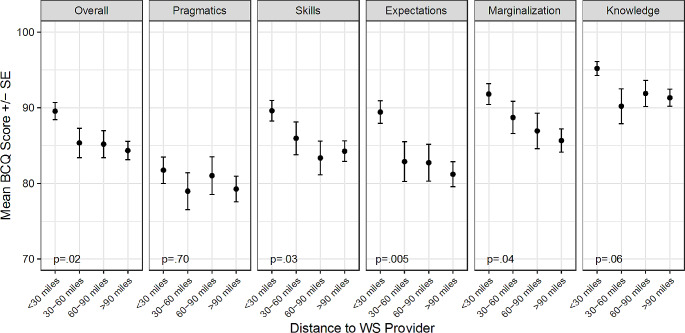
Mean BCQ overall and subscale scores by distance to WS provider

There were also associations between BCQ scores and the distance to a WS provider (Fig. 4). For the overall score (*p* = .02), the skills subscale (*p* = .03), the expectations subscale (*p* = .005), and the marginalization subscale (*p* = .04), as distance increased, mean BCQ scores decreased, indicating more barriers to care. The health care access characteristics for overall and subscale BCQ scores are available upon request.

When the factors that were correlated with either the overall BCQ score or the subscales (age, income, location, PCP knowledge of WS, distance to WS provider) were entered into multiple linear regression models, results were mostly similar to the unadjusted ANOVA results. Age remained positively associated with the pragmatics (*p* = .0004) and skills (*p* = .008) subscales. Income remained significantly associated with the overall score (*p* = .002), the skills subscale (*p* < .0001), the marginalization subscale (*p* = .04), and the knowledge and beliefs subscale (*p* = .0006). In the adjusted models, income was no longer significantly associated with the expectations subscale (*p* = .11) but it was associated with the pragmatics subscale (*p* = .03). Location remained significantly associated only with the knowledge and beliefs subscale (*p* = .04). Perceived PCP knowledge of WS was significantly associated (*p* < .05) with the overall score and all subscales except knowledge and beliefs (*p* = .09). The main change from the unadjusted ANOVA models was for distance to WS provider. This predictor was only significantly associated with the expectations subscale (*p* = .04) after adjusting for age, income, location, and PCP knowledge of WS, in contrast to the unadjusted models where it was also associated with the overall score and the skills and marginalization subscales.

## Discussion

As the first study to evaluate barriers to care in the WS population, we have identified potential areas for improving care in this unique population, as well as possible implications for individuals with similarly rare genetic syndromes. In general, our sample included caregivers who were overall satisfied with their care and experienced minimal barriers. Caregivers also felt relatively confident in their ability to explain their child’s unique needs regarding the medical aspects of their care, their child’s behavioral and emotional care, and their child’s learning and/or school-related needs. In relation to other studies that utilized the BCQ, individuals with WS had higher total BCQ scores in comparison to a generalized population of children with special healthcare needs (Seid et al. 2004) and children with sickle cell disease (Jacob et al. 2016), similar scores to children with asthma (Seid et al. 2009), and lower scores in comparison to an otolaryngology clinic population (Razdan et al., 2019) and a group of children with cleft lip and palate disorders (Bennett et al. 2018).

On the BCQ, the subscale with the lowest scores (indicating more barriers to care) was Pragmatics. In several other studies that have utilized the BCQ, this subscale also had the lowest scores, indicating logistical and cost-related issues to be significant with regards to barriers to care for a variety of special populations (Seid et al. 2009; Seid et al. 2004; Jacob et al. 2016). It is curious that neither distance to a WS provider nor income were associated with scores on the pragmatic subscale of the BCQ, it is perhaps the case that the highly-affluent, well-insured nature of our sample limited the identification of such a relationship.

Similar to the initial study that validated the BCQ, we did not identify significant differences in BCQ scores based on either race or language. Although race, language, and ethnicity are associated with a number of disparities with regards to access (Weinick et al. 2000; Yee et al. 2018; Scott and Havercamp 2014; Magaña et al. 2012), data obtained in this study supports the view that race and language could instead be viewed as markers for processes that occur during a family’s interaction with the healthcare system, rather than as a measure of the processes themselves. Data presented in this study, however, should be interpreted with caution given our relative lack of diversity in regards to race, language, and ethnicity. Our sample was largely white, affluent, and highly-educated, thus limiting our ability to detect an association between demographic characteristics other than age; it likely is the case that these lack of associations reflect limited power to identify such effects rather than a true absence of associations.

Younger age was associated with increased barriers to care for the Pragmatics and Skills subscales. These particular associations may be indicative of the challenges faced by parents of children newly-diagnosed with WS as they initially learn to navigate their child’s care, seeking medical and developmental support in the face of both logistical and cost-related issues. It would be reasonable to conclude that parents of older children, because of their experience with the healthcare system, have learned how best to navigate complex systems of care, decreasing the barriers faced.

It may be that an increased focus on early identification and early receipt of diagnosis-specific supports could have a significant impact on the care of children with WS and their families. Though this study demonstrated a lower age of diagnosis versus prior studies, our results show low enrollment in early intervention services (72%) for the 39 children ages 0–3 years in our study. In the US, early intervention is a system of coordinated services which might include physical, occupational, and speech therapy services provided at no cost for eligible children and families as federally mandated through the Individuals with Disabilities Education Act (IDEA, US Department of Education, 2017). Children with WS are likely to benefit from such services and supports given their unique developmental challenges which make specific and timely early intervention of the utmost importance (Guralnick, 2016). Formal developmental screening in the U.S. primary care setting as recommended by the American Academy of Pediatrics (Lipkin & Macias 2020), even in absence of concern for a specific diagnosis of WS, has the potential to identify children with intellectual and developmental disabilities and lead to earlier enrollment in intervention services. Should children with WS not be recognized early by their clinical phenotype or medical comorbidities, the presence of global developmental delays could prompt completion of a genetic evaluation, revealing a diagnosis of WS (Moeschler & Shevell 2014).

In comparing household incomes to BCQ scores, income was significantly associated with the overall score (*p* = .002), the expectations subscale (*p* = .04), the skills subscale (*p* = .004), the marginalization subscale (*p* = .04), and the knowledge and beliefs subscale (*p* = .0006). Lower income families may benefit from additional support when attempting to access services. Such support, which could include opportunities for care coordination services or additional opportunities for patient and caregiver education, could have the potential to aid in the reduction of income-based disparities in health outcomes for the WS population. Given that our study sample included families with relatively high incomes, further study is needed to understand in the WS population specifically what income-based health disparities might exist.

Other demographic characteristics commonly associated with increased barriers was distance to a provider that is extremely knowledgeable about WS. While increasing the number of WS specialty clinics may have the potential to reduce the aforementioned barriers to care, for many families there continues to be significant reliance on local general practitioners to provide essential and convenient care. It is reassuring to see the majority of families very confident in their PCP’s knowledge in caring for their child; however, only 15.1% of parents rate their primary care provider as being extremely knowledgeable about WS specifically. Future endeavors focused on increasing primary care knowledge of WS appears to be a necessary focus. The American Academy of Pediatrics Committee on Genetics published updated health care supervision guidelines for children with WS (Morris & Braddock 2020) that are intended for primary care providers. A previous study evaluating pediatrician adherence to health care supervision guidelines for the Down syndrome population, a relatively similar population in terms of medical complexity and developmental needs, demonstrated overall poor adherence (O’Neill et al., 2018). Future research could consider evaluating pediatrician adherence to the WS health care supervision guidelines with a goal of identifying specific opportunities for continued advocacy and education. A focus on increasing communication between larger centers with WS specialty clinics and local clinics, potentially through the use of telemedicine, could additionally be of benefit.

In the multiple linear regression models, distance to WS provider was only significantly associated with the expectations subscale after adjusting for age, income, location, and PCP knowledge of WS. As stated previously, the expectations subscale assesses parent expectations of receiving poor-quality care. The etiology of this association requires further research, but perhaps by nature of living distant from a provider with expertise in WS, caregivers are expecting to receive poor-quality care due to an expectation of local providers having poor knowledge of WS.

### Limitations

Survey respondents represented a convenience sample and were either currently enrolled in the Williams Syndrome Association Research Registry or attending the Williams Syndrome Association National Convention. These individuals may be better connected versus individuals not engaged with the Williams Syndrome Association and therefore account for the high total BCQ scores seen in our study. Approximately 50% of our participants came from either the Midwest or Northeast, areas in which there are an increased number of WS clinics as compared to the remainder of the US; however, there was not a significant difference in total BCQ scores when comparing different regions. Given that our study only included a US sample, we were also unable to access barriers to care for international individuals with WS. It should also be noted that in our sample, non-white, Hispanic, and less affluent participants were under-represented in this cohort. The survey was only distributed in English so non-English speaking families may have been unable to participate, limiting generalizability to these populations.

46% of our sample also represented parents/caregivers of individuals with WS older than 18 years old, which may decrease the strength of the finding that younger children face more barriers to care. It may also be that the BCQ does not encapsulate the barriers to care for adults with WS as the tool was developed to examine barriers to care in children, rather than adults, with special healthcare needs. It is important to note that our analysis is limited by respondent bias as all results are based upon parent perspectives. In the future, it would be important to include perspectives of individuals with WS themselves. Finally, this study was also limited by the fact that it was cross-sectional, eliminating the ability to study a temporal relationship between barriers to care and factors included in this study such as age of diagnosis and past experiences with their child’s primary care provider.

### Implications

In line with the goals of the BCQ, it is our hope that the barriers identified in this study be modifiable factors with the capacity to change. Future prospective studies, potentially using the BCQ as a process measure (Seid et al. 2009), could study the effect of such interventions. Further studies in the WS population could additionally explore primary care quality or health-related quality of life, similar to the initial study that described the BCQ (Seid et al. 2004). Although this study focused on a US-based population, it stands within reason that individuals with WS internationally may face similar barriers to care, as was seen in the study conducted by the Polish Williams Syndrome Association (Domaradzki and Walkowiak 2024). Specifically, individuals living in rural areas and younger children are likely to benefit from enhanced systems of support which emphasize not only early diagnosis, but also early access to developmental supports so as to optimize developmental outcomes.

## Conclusions

Children and adults with WS have complex medical and developmental needs. Although many caregivers report minimal barriers to care (as evidenced by higher BCQ scores), caregivers of younger children, those with lower incomes, and those living in rural areas seem to experience the most difficulties accessing care. While increasing the number of WS Specialty Clinics may have a role in improving the challenges faced by many families in this study, it is of the utmost importance that additional efforts are focused on empowering primary care providers to aid families in both their journey to diagnosis as well as ongoing medical and developmental needs. As we attempt to decrease barriers to appropriate supports and services for a wide range of individuals with intellectual and developmental disabilities, it may be pertinent for further research to unravel the etiology of the relatively high BCQ scores seen in children and adults with WS so as to identify opportunities for improving access to care for similar populations.

## Electronic supplementary material

Below is the link to the electronic supplementary material.

Supplementary Material 1

Supplementary Material 2

## Data Availability

No datasets were generated or analysed during the current study.
