# Supplementary material for: Caregiver-reported barriers to care for children and adults with Williams Syndrome
Source: J Community Genet. 2024 May 22;15(4):375–86. doi: 10.1007/s12687-024-00707-w (PMC11411038; doi:10.1007/s12687-024-00707-w)
Supplement: Supplementary file 1 — Supplementary Material 1 [file 12687_2024_707_MOESM1_ESM.docx]

**Appendix A: Barriers to Health Care Survey for Caregivers of Individuals with Williams Syndrome**

Thank you for taking the time to complete this survey. We are trying to better understand some of the barriers to healthcare for children with Williams syndrome so that we can improve the lives of our patient and families. The entire survey is confidential.

Email address:

Child’s Date of Birth:

Sex of child (circle one): M F

Race:

- White
- Hispanic or Latino
- Black or African American
- Native American or American Indian
- Asian / Pacific Islander
- Other

Primary language spoken at home:

- English
- Spanish
- French
- Arabic
- Other:

Insurance status:

- Private
- Medicaid
- Other/Self-pay
- Decline to answer

Does your child have a primary care physician? Yes No

Has your child seen his/her primary care physician in the last year? Yes No

Has your child had a successful dental exam in the last year? Yes No

Have you heard of care coordination services? Yes No

Do you utilize care coordination services? Yes No

Please rate your satisfaction with your child’s Individualized Education Plan:

- Very Satisfied
- Somewhat satisfied
- Not satisfied
- N/A (my child does not have an IEP)

Please rate your satisfaction with your child’s 504 plan:

- Very Satisfied
- Somewhat satisfied
- Not satisfied
- N/A (my child does not have a 504 plan)

What is the highest degree or level of school the mother of the child with Williams Syndrome has completed? If currently enrolled, highest degree received.

- No schooling completed
- Preschool school to 8th grade
- Some high school, no diploma
- High school graduate, diploma or the equivalent (for example: GED)
- Some college credit, no degree
- Trade/technical/vocational training
- Associate degree
- Bachelor’s degree
- Master’s degree
- Professional degree
- Doctorate degree
- Unknown/Decline to answer

What is the highest degree or level of school the father of child with Williams Syndrome has completed? If currently enrolled, highest degree received.

- No schooling completed
- Preschool to 8th grade
- Some high school, no diploma
- High school graduate, diploma or the equivalent (for example: GED)
- Some college credit, no degree
- Trade/technical/vocational training
- Associate degree
- Bachelor’s degree
- Master’s degree
- Professional degree
- Doctorate degree
- Unknown/Decline to answer

If the primary caregiver is not the mother or father, please answer the following. Otherwise, leave blank. What is the highest degree or level of school the primary caregiver of child with Williams Syndrome has completed? If currently enrolled, highest degree received.

Please indicate relationship of primary caregiver:

- No schooling completed
- Preschool to 8th grade
- Some high school, no diploma
- High school graduate, diploma or the equivalent (for example: GED)
- Some college credit, no degree
- Trade/technical/vocational training
- Associate degree
- Bachelor’s degree
- Master’s degree
- Professional degree
- Doctorate degree
- Unknown/Decline to answer

Please approximate your total annual household income

- Less than $20,000
- $20,000 to $34,999
- $35,000 to $49,999
- $50,000 to $74,999
- $75,000 to $99,999
- Over $100,000
- Decline to answer

Do you currently receive Supplemental Security Income (SSI?)

- Yes
- No
- Decline to answer

Are you connected to a board of Developmental Disabilities?

- Yes
- No
- Decline to answer

Which region of the country do you currently live in?

- Midwest - IA, IL, IN, KS, MI, MN, MO, ND, NE, OH, SD, WI
- Northeast - CT, DC, DE, MA, MD, ME, NH, NJ, NY, PA, RI, VT
- Southeast - AL, AR, FL, GA, KY, LA, MS, NC, SC, TN, VA, WV
- Southwest - AZ, NM, OK, TX
- West - AK, CA, CO, HI, ID, MT, NV, OR, UT, WA, WY

Which of the following best describes the area you live in?

- Urban
- Suburban
- Rural

How old was your child when he/she was diagnosed with Williams Syndrome?

___ months or ___years

How was your child’s diagnosis of Williams Syndrome confirmed?

- FISH
- Microarray
- Clinical diagnosis (diagnosis made by physician based on facial or behavioral features)
- Other:

How would you describe your primary care provider’s knowledge about Williams Syndrome?

- Extremely knowledgeable
- Somewhat knowledgeable
- Limited knowledge
- My primary care provider has no knowledge of Williams Syndrome

How confident are YOU explaining the following to a healthcare provider unfamiliar with Williams Syndrome? (check box that applies)

|  | Very confident | Somewhat Confident | Not confident at all |
| --- | --- | --- | --- |
| Overall medical aspects of your child’s care |  |  |  |
| Behavioral/emotional aspects of your child’s care |  |  |  |
| Learning/school-related aspects of your child’s care |  |  |  |

Please check the box next to the medical aspects of your child’s care with which you are most concerned (check all that apply):

- Cardiovascular (Heart)
- Renal (Kidney)
- Musculoskeletal
- Endocrine
- Neurology (ex: Chiari malformation, tremors)
- Gastrointestinal
- Hearing
- Vision
- Behavioral/Mental Health

Please estimate your distance to a provider that is extremely knowledgeable about Williams Syndrome

- <30 miles
- 30-60 miles
- 60-90 miles
- >90 miles

Please estimate your distance to a Pediatric Emergency Room

- <30 miles
- 30-60 miles
- 60-90 miles
- >90 miles

Current therapies through Early Intervention Services (0-3 years, check all that apply):

- Speech therapy
  - Time Spent: minutes per week/month (circle one)
- Occupational Therapy
  - Time Spent: minutes per week/month (circle one)
- Physical Therapy
  - Time Spent: minutes per week/month (circle one)
- My child receives no Early Intervention Services

Current School-based therapies (check all that apply):

- Speech therapy
  - Time Spent: minutes per week/month (circle one)
- Occupational Therapy
  - Time Spent: minutes per week/month (circle one)
- Physical Therapy
  - Time Spent: minutes per week/month (circle one)
- Music Therapy
  - Time Spent: minutes per week/month (circle one)
- My child is not yet in school
- My child does not receive additional therapies at school
- My child is an adult

Current private/outpatient therapies(check all that apply):

- Speech therapy
  - Time Spent: minutes per week/month (circle one)
  - Distance to location of therapy: miles
- Occupational Therapy
  - Time Spent: minutes per week/month (circle one)
  - Distance to location of therapy: miles
- Physical Therapy
- Time Spent: minutes per week/month (circle one)
  - Distance to location of therapy: miles
- Music Therapy
  - Time Spent: minutes per week/month (circle one)
  - Distance to location of therapy: miles
- None

Does your child have a diagnosis of Anxiety? Yes No

-If yes, what was their age at time of diagnosis?

-If yes, is he/she participating in other therapies for management of Anxiety? (check all that apply)

- Cognitive Behavioral Therapy
- Stress/relaxation therapy
- Yoga/Meditation
- Other:

Does your child have a diagnosis of ADHD? Yes No

-If yes, what was their age at time of diagnosis?

-If yes, is he/she participating in other therapies for management of ADHD? (check all that apply)

- Behavior therapy
- Neurofeedback
- Parent training
- Other:

What is your biggest struggle in obtaining medical care for your child with Williams Syndrome?

**Barriers to Care Questionnaire**

Parents often face barriers when trying to get health care for their children. We are interested in the kinds of things that interfere with getting health care for your child(ren). Please check the box that is most accurate next to each area that may or may not be a barrier to your child’s care.

|  | *No Problem* | *Small Problem* | *Problem* | *Big Problem* | *Very Big Problem* |
| --- | --- | --- | --- | --- | --- |
| *Knowing How to Make the health care system work for you* |  |  |  |  |  |
| *Doctors or nurses not fluent in your language* |  |  |  |  |  |
| *Doctors who speak in a way that is too technical or medical* |  |  |  |  |  |
| *Getting referrals to specialists* |  |  |  |  |  |
| *Understanding doctor’s orders* |  |  |  |  |  |
| *Having enough information about how the health care system works* |  |  |  |  |  |
| *Needing to be more “savvy” or knowledgeable about getting health care* |  |  |  |  |  |
| *Getting enough help with paperwork or forms* |  |  |  |  |  |
| *Feeling like doctors are trying to give as little service as possible* |  |  |  |  |  |
| *Feeling like the health care system is trying to give as little service as possible* |  |  |  |  |  |
| *Impatient doctors* |  |  |  |  |  |
| *Intimidating doctors* |  |  |  |  |  |
| *Rude office staff* |  |  |  |  |  |
| *Uncaring office staff* |  |  |  |  |  |
| *Getting the doctor to listen to you* |  |  |  |  |  |
| *Getting your questions answered* |  |  |  |  |  |
| *Not knowing what to expect from one visit to the next* |  |  |  |  |  |
| *Being judged on your appearance, your ancestry, or your accent* |  |  |  |  |  |
| *Doctors rushing you and your child through the visit* |  |  |  |  |  |
| *Offices and staff that are not child-friendly* |  |  |  |  |  |
| *Mistakes made by doctors or nurses* |  |  |  |  |  |
| *Worrying that doctors and nurses will not do what is right for your child* |  |  |  |  |  |
| *Doctors treating the symptom without finding out the cause of the illness* |  |  |  |  |  |
| *Getting a thorough examination* |  |  |  |  |  |
| *Lack of communication between my child’s doctor and others in the health care system* |  |  |  |  |  |
| *Lack of communication between different parts of the health care system* |  |  |  |  |  |
| *Disagreeing with the doctor’s orders* |  |  |  |  |  |
| *Doctors not believing in home or traditional remedies* |  |  |  |  |  |
| *Doctors giving you instructions that seem wrong* |  |  |  |  |  |
| *Doctors or nurses that have different ideas about health than you do* |  |  |  |  |  |
| *Getting to the doctor’s office* |  |  |  |  |  |
| *Getting hold of the doctor’s office or clinic by telephone* |  |  |  |  |  |
| *Having to wait too many days for an appointment* |  |  |  |  |  |
| *Getting care after hours or on the weekends* |  |  |  |  |  |
| *Having to take care of household responsibilities* |  |  |  |  |  |
| *Having to take time off work* |  |  |  |  |  |
| *Having to wait too long in the waiting room* |  |  |  |  |  |
| *Meeting the needs of other family members* |  |  |  |  |  |
| *The cost of health care* |  |  |  |  |  |

Please share any additional information in response to the questions above:
