# Supplementary material for: Caregiver-reported barriers to care for children and adults with Williams Syndrome
Source: J Community Genet. 2024 May 22;15(4):375–86. doi: 10.1007/s12687-024-00707-w (PMC11411038; doi:10.1007/s12687-024-00707-w)
Supplement: Supplementary file 2 — Supplementary Material 2 [file 12687_2024_707_MOESM2_ESM.docx]

**Appendix B. Supplemental Data**

S**upplemental Table B.1:** Mean BCQ scores by levels of all demographics

|  |  |  | **Overall** | |  | **Pragmatics** | |  | **Skills** | |
| --- | --- | --- | --- | --- | --- | --- | --- | --- | --- | --- |
| **Characteristic** | **Level** | **N** | **Mean (SD)** | **P-value** |  | **Mean (SD)** | **P-value** |  | **Mean (SD)** | **P-value** |
| CG Education | HS or less | 21 | 83.1 (17.8) | 0.26 |  | 80.2 (17.9) | 0.75 |  | 80.2 (18.8) | 0.19 |
| (highest in HH) | Some college/Associates/Trade/Voc | 65 | 84.4 (15.4) |  |  | 78.8 (19.0) |  |  | 85.4 (15.7) |  |
|  | Bachelor's degree | 110 | 86.4 (11.7) |  |  | 80.3 (16.8) |  |  | 86.0 (15.1) |  |
|  | Master's degree | 88 | 88.4 (10.7) |  |  | 82.5 (17.0) |  |  | 87.9 (13.2) |  |
|  | Professional/Doctoral | 34 | 86.6 (10.9) |  |  | 79.3 (17.7) |  |  | 89.0 (9.8) |  |
| HH Income | Less than $50,000 | 41 | 82.8 (14.9) | **0.002** |  | 78.6 (19.5) | 0.06 |  | 81.9 (17.4) | **0.004** |
|  | $50,000 to $74,999 | 57 | 83.6 (13.9) |  |  | 77.4 (18.1) |  |  | 83.0 (15.2) |  |
|  | $75,000 to $99,999 | 50 | 83.8 (15.2) |  |  | 76.5 (18.6) |  |  | 84.6 (15.9) |  |
|  | Over $100,000 | 133 | 89.2 (9.9) |  |  | 83.1 (15.9) |  |  | 89.3 (12.1) |  |
| Insurance Type | Medicaid | 100 | 86.6 (13.4) | 0.50 |  | 82.9 (17.2) | 0.28 |  | 86.7 (15.2) | 0.97 |
|  | Private | 191 | 86.7 (11.9) |  |  | 79.6 (17.5) |  |  | 86.3 (14.1) |  |
|  | Other/Self-Pay | 20 | 83.2 (16.2) |  |  | 78.6 (18.4) |  |  | 86.6 (14.6) |  |
| Location | Urban | 48 | 89.4 (10.1) | 0.07 |  | 83.3 (14.6) | 0.28 |  | 90.1 (11.0) | 0.14 |
|  | Suburban | 198 | 86.4 (13.1) |  |  | 80.6 (17.6) |  |  | 85.5 (16.1) |  |
|  | Rural | 69 | 83.9 (13.0) |  |  | 78.1 (18.7) |  |  | 86.1 (11.8) |  |
| Race | White | 262 | 86.7 (11.8) | 0.23 |  | 81.2 (16.4) | 0.10 |  | 86.8 (13.5) | 0.19 |
|  | Non-White | 55 | 84.5 (16.3) |  |  | 76.9 (21.5) |  |  | 84.0 (19.0) |  |
| Region | Midwest | 75 | 86.3 (13.8) | 0.35 |  | 81.0 (18.9) | 0.53 |  | 87.3 (13.0) | 0.21 |
|  | Northeast | 91 | 88.5 (11.3) |  |  | 82.1 (15.3) |  |  | 87.8 (13.4) |  |
|  | Southeast | 59 | 84.2 (12.9) |  |  | 79.6 (17.3) |  |  | 82.9 (16.6) |  |
|  | Southwest | 31 | 85.5 (15.9) |  |  | 82.4 (16.7) |  |  | 83.7 (19.0) |  |
|  | West | 52 | 86.6 (10.8) |  |  | 77.2 (19.6) |  |  | 87.6 (13.6) |  |
| Sex | Female | 167 | 86.7 (12.2) | 0.48 |  | 81.0 (16.6) | 0.49 |  | 87.3 (14.5) | 0.17 |
| (WS individual) | Male | 148 | 85.7 (13.3) |  |  | 79.6 (18.4) |  |  | 85.1 (14.7) |  |

P-values from one-way ANOVA

**Supplemental Table B.1 (con’t)**

|  |  |  | **Expectations** | |  | **Marginalization** | |  | **Knowledge and Beliefs** | |
| --- | --- | --- | --- | --- | --- | --- | --- | --- | --- | --- |
| **Characteristic** | **Level** | **N** | **Mean (SD)** | **P-value** |  | **Mean (SD)** | **P-value** |  | **Mean (SD)** | **P-value** |
| CG Education | HS or less | 21 | 81.3 (25.1) | 0.32 |  | 83.4 (25.2) | 0.20 |  | 90.5 (14.6) | 0.22 |
| (highest in HH) | Some college/Associates/Trade/Voc | 65 | 81.0 (21.4) |  |  | 86.2 (17.8) |  |  | 90.3 (15.1) |  |
|  | Bachelor's degree | 110 | 84.9 (15.8) |  |  | 88.5 (13.0) |  |  | 92.2 (12.3) |  |
|  | Master's degree | 88 | 86.5 (14.4) |  |  | 91.0 (11.9) |  |  | 94.3 (10.1) |  |
|  | Professional/Doctoral | 34 | 82.4 (15.8) |  |  | 87.7 (17.9) |  |  | 94.7 (7.4) |  |
| HH Income | Less than $50,000 | 41 | 78.8 (22.7) | **0.04** |  | 85.1 (16.8) | **0.04** |  | 89.5 (13.4) | **0.0006** |
|  | $50,000 to $74,999 | 57 | 82.8 (18.0) |  |  | 85.3 (18.6) |  |  | 89.5 (14.2) |  |
|  | $75,000 to $99,999 | 50 | 81.6 (19.5) |  |  | 86.8 (17.0) |  |  | 89.5 (15.5) |  |
|  | Over $100,000 | 133 | 86.9 (14.2) |  |  | 91.0 (12.3) |  |  | 95.7 (9.2) |  |
| Insurance Type | Medicaid | 100 | 83.3 (19.4) | 0.17 |  | 87.8 (16.4) | 0.39 |  | 92.4 (11.6) | 0.28 |
|  | Private | 191 | 85.1 (15.5) |  |  | 89.2 (15.0) |  |  | 93.2 (11.6) |  |
|  | Other/Self-Pay | 20 | 77.7 (24.2) |  |  | 84.5 (15.0) |  |  | 88.8 (18.8) |  |
| Location | Urban | 48 | 86.7 (17.9) | 0.19 |  | 92.3 (10.6) | 0.11 |  | 94.7 (9.0) | **0.003** |
|  | Suburban | 198 | 84.3 (17.3) |  |  | 87.9 (16.4) |  |  | 93.4 (10.5) |  |
|  | Rural | 69 | 80.9 (17.8) |  |  | 86.3 (15.4) |  |  | 88.1 (17.1) |  |
| Race | White | 262 | 84.5 (16.3) | 0.28 |  | 88.8 (13.9) | 0.17 |  | 92.2 (12.6) | 0.29 |
|  | Non-White | 55 | 81.7 (22.3) |  |  | 85.7 (21.4) |  |  | 94.1 (10.2) |  |
| Region | Midwest | 75 | 83.4 (19.2) | 0.45 |  | 88.2 (15.7) | 0.37 |  | 91.8 (12.2) | 0.21 |
|  | Northeast | 91 | 86.9 (15.0) |  |  | 90.6 (15.8) |  |  | 95.0 (10.4) |  |
|  | Southeast | 59 | 82.0 (17.1) |  |  | 85.8 (16.7) |  |  | 90.5 (13.7) |  |
|  | Southwest | 31 | 82.8 (20.6) |  |  | 86.4 (18.3) |  |  | 92.3 (16.7) |  |
|  | West | 52 | 85.4 (15.4) |  |  | 89.5 (11.0) |  |  | 93.3 (9.7) |  |
| Sex | Female | 167 | 83.9 (17.5) | 0.99 |  | 88.3 (16.0) | 0.95 |  | 93.1 (11.1) | 0.32 |
| (WS individual) | Male | 148 | 83.9 (17.6) |  |  | 88.2 (14.9) |  |  | 91.8 (13.4) |  |

P-values from one-way ANOVA

**Supplemental Table B.2:** Mean BCQ overall and subscale scores by factors related to health care access

|  |  |  | **Overall** | | |  | **Pragmatics** | |  | **Skills** | | |
| --- | --- | --- | --- | --- | --- | --- | --- | --- | --- | --- | --- | --- |
| **Characteristic** | **Level** | **N** | **Mean (SD)** | **P-value** | |  | **Mean (SD)** | **P-value** |  | **Mean (SD)** | **P-value** | |
| PCP's Knowledge of WS | Extremely knowledgeable | 47 | 90.0 (11.0) | **<.0001** | |  | 84.4 (14.9) | **0.005** |  | 92.2 (8.8) | **0.0003** | |
|  | Somewhat knowledgeable | 143 | 86.9 (11.5) |  | |  | 79.8 (17.4) |  |  | 86.8 (13.2) |  | |
|  | Limited knowledge | 112 | 86.0 (12.5) |  | |  | 81.6 (17.2) |  |  | 84.7 (15.8) |  | |
|  | No knowledge | 16 | 73.4 (19.8) |  | |  | 67.0 (20.2) |  |  | 75.2 (21.8) |  | |
| Distance to Pediatric ER | <30 miles | 226 | 87.4 (11.9) | 0.054 | |  | 81.7 (16.6) | 0.10 |  | 87.0 (14.3) | 0.36 | |
|  | 30-60 miles | 46 | 83.0 (14.3) |  | |  | 76.2 (17.4) |  |  | 83.7 (15.3) |  | |
|  | 60-90 miles | 17 | 82.8 (16.0) |  | |  | 77.0 (22.2) |  |  | 83.8 (18.0) |  | |
|  | >90 miles | 23 | 83.1 (12.6) |  | |  | 75.5 (20.1) |  |  | 83.6 (13.9) |  | |
| Distance to WS Provider | <30 miles | 93 | 89.6 (11.0) | **0.02** | |  | 81.8 (16.8) | 0.70 |  | 89.6 (13.1) | **0.03** | |
|  | 30-60 miles | 53 | 85.4 (14.2) |  | |  | 79.0 (17.7) |  |  | 86.0 (15.8) |  | |
|  | 60-90 miles | 47 | 85.2 (12.2) |  | |  | 81.0 (17.0) |  |  | 83.4 (15.1) |  | |
|  | >90 miles | 116 | 84.3 (13.2) |  | |  | 79.3 (18.1) |  |  | 84.3 (14.7) |  | |
|  |  |  | **Expectations** | | |  | **Marginalization** | |  | **Knowledge and Beliefs** | | |
| **Characteristic** | **Level** | **N** | **Mean (SD)** | | **P-value** |  | **Mean (SD)** | **P-value** |  | **Mean (SD)** | | **P-value** |
| PCP's Knowledge of WS | Extremely knowledgeable | 47 | 87.7 (17.4) | | **0.0006** |  | 91.5 (14.3) | **0.0001** |  | 94.1 (12.0) | | **0.03** |
|  | Somewhat knowledgeable | 143 | 85.7 (15.2) | |  |  | 89.6 (12.9) |  |  | 92.8 (12.6) | |  |
|  | Limited knowledge | 112 | 83.1 (16.7) | |  |  | 87.8 (15.2) |  |  | 92.7 (11.0) | |  |
|  | No knowledge | 16 | 68.4 (28.5) | |  |  | 72.4 (28.4) |  |  | 84.0 (15.0) | |  |
| Distance to Pediatric ER | <30 miles | 226 | 85.5 (16.6) | | 0.11 |  | 89.5 (14.9) | 0.16 |  | 93.4 (10.9) | | 0.13 |
|  | 30-60 miles | 46 | 79.9 (18.6) | |  |  | 85.7 (17.4) |  |  | 89.7 (16.2) | |  |
|  | 60-90 miles | 17 | 79.8 (21.6) | |  |  | 84.2 (16.3) |  |  | 89.0 (16.5) | |  |
|  | >90 miles | 23 | 81.2 (16.4) | |  |  | 84.2 (16.4) |  |  | 90.8 (11.3) | |  |
| Distance to WS Provider | <30 miles | 93 | 89.4 (14.5) | | **0.005** |  | 91.8 (13.2) | **0.04** |  | 95.2 (8.9) | | 0.06 |
|  | 30-60 miles | 53 | 82.9 (19.2) | |  |  | 88.7 (15.5) |  |  | 90.2 (16.7) | |  |
|  | 60-90 miles | 47 | 82.7 (16.7) | |  |  | 86.9 (16.2) |  |  | 91.9 (11.9) | |  |
|  | >90 miles | 116 | 81.2 (17.9) | |  |  | 85.7 (16.6) |  |  | 91.3 (12.3) | |  |

P-values from one-way ANOVA

**Supplemental Table B.3:** Services received, individuals aged 18 and younger only (sample sizes shown in table body)

| **Therapies Received*** | | **% (N)** |
| --- | --- | --- |
| Early Intervention Services (ages 0-3) | |  |
|  | Speech therapy | 56% (22/39) |
|  | Occupational therapy | 56% (22/39) |
|  | Physical therapy | 56% (22/39) |
|  | All three endorsed | 38% (15/39) |
|  | At least one endorsed | 72% (28/39) |
|  | None of the three endorsed | 28% (11/39) |
| School-based Therapies (ages 3-18) | |  |
|  | Speech therapy | 76% (102/134) |
|  | Occupational therapy | 75% (100/134) |
|  | Physical therapy | 48% (64/134) |
|  | Music therapy | 11% (15/134) |
|  | All four endorsed | 8% (11/134) |
|  | At least one endorsed | 86% (115/134) |
|  | None of these four endorsed | 14% (19/134) |
| Private/Outpatient Therapies (ages 0-18) | |  |
|  | Speech therapy | 23% (37/162) |
|  | Occupational therapy | 25% (40/162) |
|  | Physical therapy | 15% (25/162) |
|  | Music therapy | 6% (10/162) |
|  | All four endorsed | 1% (2/162) |
|  | At least one endorsed | 40% (65/162) |
|  | None of these four endorsed | 60% (97/162) |
